# Sintering, Microstructure, and Mechanical Properties of TiTaNbZrHf High-Entropy Alloys Prepared by Cold Isostatic Pressing and Pressure-Less Sintering of Hydrides

**DOI:** 10.3390/ma16051759

**Published:** 2023-02-21

**Authors:** Yubing Chen, Peidong Liu, Zhaowang Dong, Hanning Liu, Junjie Wang, Xueyi Guo, Yang Xia, Qinmeng Wang

**Affiliations:** 1 School of Metallurgy and Environment, Central South University, Changsha 410083, China; 2 National and Regional Joint Engineering Research Center of Nonferrous Metal Resources Recycling, Changsha 410083, China

**Keywords:** powder metallurgy, refractory high-entropy alloys, powder size, element diffusion, high mechanical properties

## Abstract

A TiTaNbZrHf refractory high-entropy alloy (RHEA) was synthesized through a cold isostatic pressing and a pressure-less sintering process in a hydrogen atmosphere using a powder mixture of metal hydride prepared either by mechanical alloying (MA) or by rotating mixing. This study investigates how differences in powder particle sizes impact the RHEA’s microstructure and mechanical properties. HCP (a = b = 3.198 Å, c = 5.061 Å) and BCC2 (a = b = c = 3.40 Å) phases were observed in the microstructure of coarse powder TiTaNbZrHf RHEAs at 1400 °C. In contrast, fine powder RHEAs were found to possess two-phase structures of HCP and BCC1 (a = b = c = 3.36 Å) with a higher hardness of 431 HV, compression strength of 1620 MPa, and a plasticity of >20%.

## 1. Introduction

High-entropy alloys (HEAs) are composed of five or more alloying elements, each of which is composed of equal or nearly equal atomic ratios. [1,2]. HEAs differ from traditional alloys because of high-entropy effects, lattice distortion effects, kinetic delayed diffusion effects, and “cocktail” effects [3]. They can easily obtain good-thermal-stability solid solution phase, nanostructures, and even amorphous structures. HEAs have attracted much attention owing to their high strength [4], high hardness [5], high corrosion resistance [6], high wear resistance [7], etc. 

Refractory high-entropy alloys (RHEAs) are mostly composed of refractory elements with melting points higher than 1800 °C. RHEAs can maintain high strength (>400 MPa) at 1600 °C, making them an ideal material to replace nickel-based superalloys, whose maximum working temperature is approximately 1200 °C [8]. In particular, TiTaNbZrHf RHEAs combine desirable refractory properties with light weight [7,8], whereby the density is below 10 g/cm^3^. TiTaNbZrHf RHEAs possess good mechanical properties [9], corrosion resistance [6], hydrogen absorption properties [10] and good biocompatibility [11]. Furthermore, these are the only ductile RHEAs at room temperature [12].

The method traditionally used on industrial scales for preparing HEAs is the vacuum melting method [13]. TiTaNbZrHf alloys are prepared by this method with a high yield strength (about 890 MPa) [14] and high tensile plasticity of 28% at extremely low temperatures [15]. However, the high melting point of refractory metal elements for vacuum melting requires high-temperature preparation [16]. Therefore, the development of RHEAs has been hindered by microstructure defects caused by vacuum melting, shrinkage cavity, shrinkage porosity, and segregation [16,17].

The powder metallurgy (PM) method for preparing HEAs generally uses metal powders as raw materials and includes the steps of ball milling, pressing, sintering, and post-processing. PM has the advantages of low temperature sintering, avoiding segregation, dendrite formation, polycrystalline precipitation, and high material utilization [18]. Other standard methods for the preparation of HEAs include mechanical alloying (MA), hot isostatic pressing, and spark plasma sintering (SPS) [4,19,20]. For example, TiNbTa_0.5_ZrAl_0.5_ with BCC single-phase, uniform composition distribution, and no segregation was prepared by PM and spark plasma sintering [21]. This TiNbTa_0.5_ZrAl_0.5_ possessed a compressive yield strength of 1500 MPa, and a high compressive strain at room temperature of 18%. Another TiTaNbZrHf [22] alloy had a hardness of up to 584 HV, but had a very high oxygen content of about 1.07 wt% that can be attributed to the MA and SPS processes. Thus, the current PM method for preparing HEAs still has some drawbacks. Such drawbacks include long-term ball milling of HEAs to obtain small-size powder particles that can lead to oxygen pollution [23], residual voids that result in reduced mechanical properties and the subsequent need for further heat treatment [24,25], a long sintering process, higher energy consumption, and so on.

Studies have shown that adding metal hydride enables a sintered alloy to have a higher density and grain size and better mechanical properties than pure metal powders [26]. In addition, metal hydride powders used in preparing HEAs can reduce the sintering temperature and prepare aggregate blocks with high hardness and phase stability [23].

This work used hydride powders as raw materials to prepare TiTaNbZrHf alloys by PM. The microstructure and mechanical properties of the alloys were then observed after cold pressing and sintering by different particle sizes and sintering temperatures.

## 2. Experimental Procedures

TiTaNbZrHf alloy powders were prepared by PM and mixing metal hydride powders (with a purity of 99%), such as TiH_2_, TaH_2_, NbH_2_, ZrH_2,_ and HfH_2_. The D_(5,0)_ particle sizes of TaH_2_, NbH_2_, ZrH_2,_ and HfH_2_ were 18.38 μm, 48.03 μm, 30.22 μm, and 9.57 μm, respectively. The particle sizes of TiH_2_ were 5–8 μm, 150 mesh (about 100 μm), and 300 mesh (about 48 μm), respectively. The raw powders at equiatomic ratio were blended in a Planetary Mill blender (Nanjing University Machinery Factory) for 0.5 h to 2 h at 580 rpm and a ball-to-powder ratio of 5:1. The alloy powders were named HEA-0.5, HEA-1, HEA-2, corresponding to ball milling times of 5–8 μm TiH_2_ for 0.5 h, 1 h, 2 h, respectively. The 150 mesh and 300 mesh TiH_2_ were named HEA-150 and HEA-300, respectively. The powder mixture was compressed via cold isostatic pressing at 350 MPa.

The sintering temperatures for the mixed atmosphere of H_2_ and Ar were 1000 °C, 1100 °C, 1200 °C, 1300 °C, and 1400 °C. The compressed powder mixtures were heated to 800 °C within 80 min at a heating rate of 10 °C min^−1^, and from 800 °C to the desired sintering temperature at a heating rate of 5 °C min^−1^ in an H_2_ atmosphere, and followed by 2 h of holding time at the sintering temperature. After the mixtures were held at the sintering temperature for 2 h, they were transferred to an Ar atmosphere until the sintering was complete and cooled down to 30–50 °C. 

The phase composition of the synthesized alloys was investigated by X-ray diffraction (XRD) using Cu Kα radiation. A laser particle size analyzer measured the size distributions of the mechanically alloyed powder. The microstructures of the alloys and powders were observed by scanning electron microscopy (SEM) and energy-dispersive X-ray spectrometry (EDS). Transmission Electron Microscopy (TEM) was used to image the fine-scale microstructural features, followed by a higher-resolution analysis of the resultant TEM images. The density of the alloys was determined in distilled water using Archimedes’ method. Microhardness measurements were taken using a Vickers hardness tester, and an average of five measurements was reported. Compressive stress–strain testing was conducted at room temperature using a universal testing machine with the sintered block cylinders (5 × 15 mm) at an engineering strain rate of 10^−3^ s^–1^. 

## 3. Results and Discussion

### 3.1. HEA Powders

**Figure 1:**
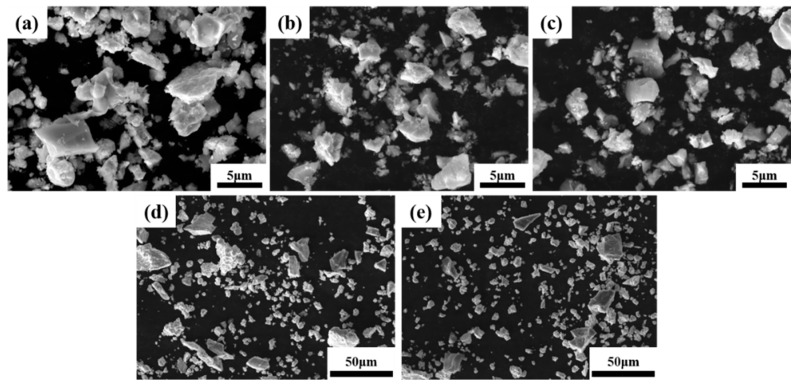
Morphology of HEA powders observed by SEM: (**a**) HEA-0.5; (**b**) HEA-1; (**c**) HEA-2; (**d**) HEA-150; (**e**) HEA-300.

**Figure 2:**
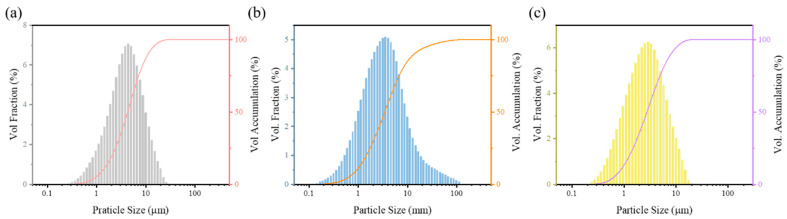
Particle size distribution of HEA powders: (**a**) HEA-0.5; (**b**) HEA-1; (**c**) HEA-2.

Figure 1 shows SEM images of the microstructure of TiTaNbZrHf HEA powders after different milling times using different methods. As shown in Figure 1, the size of powders after MA decreases with increasing milling time, demonstrating an irregular shape. After ball milling, due to the brittleness of metal hydride, very small powder can be obtained. The D_(5,0)_ values of the HEA-0.5, HEA-1, and HEA-2 powders were 4.282, 3.445, and 2.759 μm, respectively, as shown in Figure 2. These D_(5,0)_ values were smaller than the particle size of each raw material powder. After rotating mixing, the shape and particle size of the HEA powders were similar to the raw material powders, wherein the larger particles were approximately 30–50 μm, and the smaller particles were approximately 10 μm.

### 3.2. Phase and Microstructure of TiTaNbZrHf HEA 

**Figure 3:**
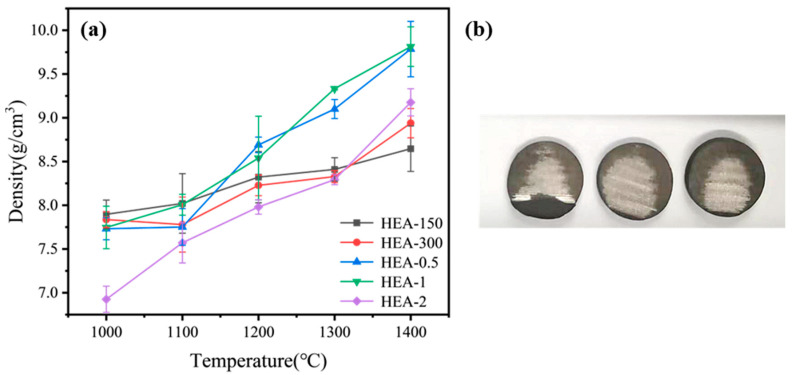
(**a**) Density of TiTaNbZrHf alloys at different temperatures; (**b**) HEA-2 alloy profile at 1000 °C.

The density of all alloys was tested using the Archimedes method, and the results are reported in Figure 3a. Figure 3a shows that the particle size of the powder is correlated with the density of the alloys. Apart from HEA-2, all samples showed that a smaller HEA particle size resulted in a lower density. In particular, at 1000 °C, the significantly lower density of HEA-2 may be attributed to incomplete sintering of the alloy. Incomplete sintering of the HEA-2 alloy was evident from the appearance of a metallic luster only in the central area of the alloy (Figure 3b). With increasing sintering temperature, the sample density also increased. With a shorter ball milling time, a larger particle size, and a sintering temperature of 1400 °C, the density of HEA-2 was higher than those of HEA-150 and HEA-300 but lower than those of HEA-0.5 and HEA-1. At 1400 °C, the density of HEA-0.5 and HEA-1 reached 9.46 g/cm^3^ and 9.831 g/cm^3^, respectively. These density values were highly similar to the theoretical value of 9.9 g/cm^3^ reported in the literature [27,28]. The comparison between the test density of the alloy and the theoretical density reflects the quality of the prepared alloy to a certain extent. The density of HEA-1 was calculated at 99.3% of the theoretical density, indicating that the HEA-1 at 1400 °C is highly dense with minimal voids.

**Figure 4:**
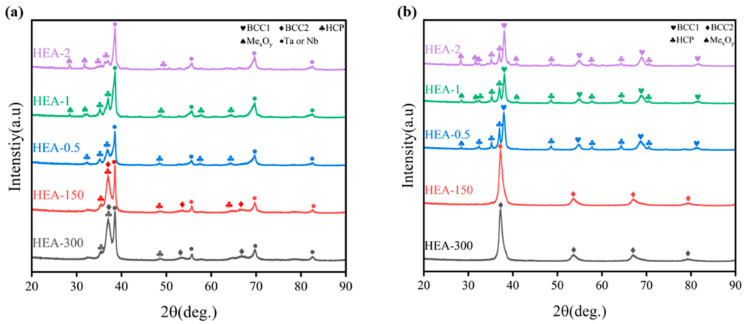
XRD patterns of TiTaNbZrHf HEAs at different temperatures: (**a**) XRD patterns of TiTaNbZrHf HEAs at 1000 °C; (**b**) XRD patterns of TiTaNbZrHf HEAs at 1400 °C.

The XRD results for the samples at 1000 °C and 1400 °C are shown in Figure 4. When sintered at 1000 °C, all alloys showed multiphase structures. HCP (a = b = 3.198Å, c = 5.061Å) phase and characteristic peaks of Ta or Nb were also observed in all the alloys. The BCC2 (a = b = c = 3.40Å) phase appeared in HEA-300 and HEA-150, while the new Me_x_O_y_ phase appeared in alloys such as HEA-0.5, HEA-1, and HEA-2 after ball milling. With increasing sintering temperature, the XRD pattern changed significantly at 1400 °C. Particularly, the XRD patterns of HEA-150 and HEA-300 changed from a multi-phase structure to a BCC2 single-phase structure. HEA-0.5, HEA-1, and HEA-2 exhibited a dual-phase structure of HCP and BCC1 (a = b = c = 3.36 Å), and the Me_x_O_y_ phase was also combined. TiTaNbZrHf HEA displayed a stable BCC single-phase structure [8,29,30,31]. However, the alloys prepared by PM presented multiphase structures like BCC and HCP phase [23,27], which was similar to the results of our experiment. This could be attributed to different element migration rates caused by the powder particle size, as discussed in detail below.

**Figure 5:**
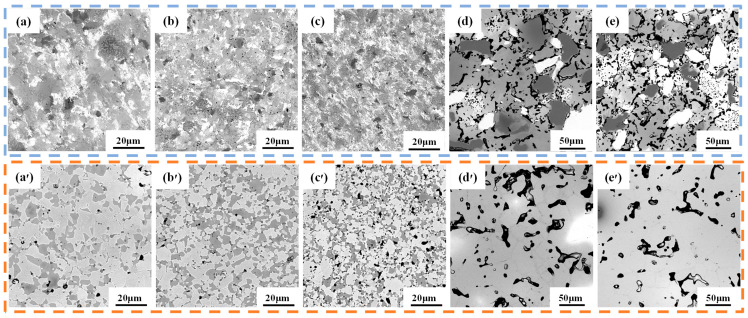
SEM images of as-sintered TiTaNbZrHf HEAs with different particle size powders at different temperatures: (**a**–**e**) HEA-0.5, HEA-1, HEA-2, HEA-150, HEA-300 at 1000 °C; (**a′**–**e′**) HEA-0.5, HEA-1, HEA-2, HEA-150, HEA-300 at 1400 °C.

**Figure 6:**
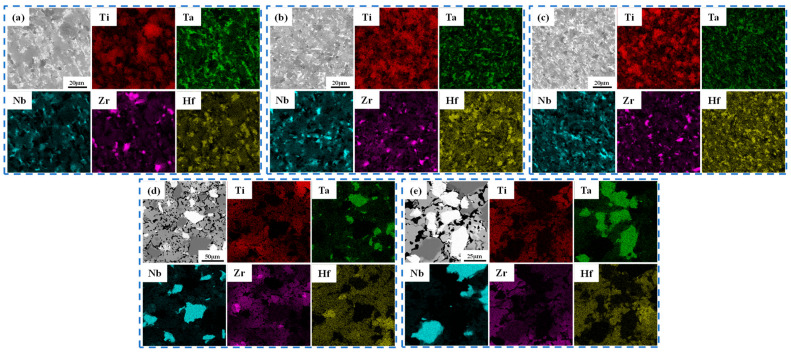
Elemental distributions of the TiNbTaZrHf HEAs at 1000 °C: (**a**) HEA-0.5; (**b**) HEA-1; (**c**) HEA-2; (**d**) HEA-150; (**e**) HEA-300.

Figure 5a–e illustrate the microstructures of the alloys at different powder particle sizes and sintering temperatures. After sintering at 1000 °C, the microstructures of HEA-1, HEA-0.5, and HEA-2 appeared highly disordered, and the alloy phase was not apparent. However, HEA-150 and HEA-300 demonstrated prominent multiphase structures. Both elemental distributions and chemical compositions of TiTaNbZrHf HEAs were examined by EDS (Figure 6), wherein obvious element aggregations were observed. In the HEA-150 and HEA-300 microstructures, the black phase indicates the aggregation of Nb, the bright white phase is the aggregation of Ta, and the gray phase is composed of Ti, Zr, and Hf. The phase composed of HEA-0.5, HEA-1, and HEA-2 is not apparent, and each element has a slight aggregation, similar to HEA-150 and HEA-300. The bright white parts represent Ta and Nb, and the dark areas are Zr and Hf. Thus, the XRD pattern of low-temperature sintering shows characteristic peaks of Ta or Nb and HCP phases formed by Zr and Hf.

After higher-temperature sintering (1400 °C), the aggregation of bulk elements in HEA-150 and HEA-300 disappeared. In addition, the light and dark phases previously observed at a lower sintering temperature (1000 °C) did not appear. The absence of light and dark phases combined with the XRD results meant that the alloys formed a single BCC2 phase. However, freaked pores were present in the alloy. Furthermore, a small number of circular pores were observed. The number of pores in the alloy HEA-300 decreased as the density increased. SEM images also revealed that ball milling duration had an appreciable effect on the alloys’ microstructure. For instance, HEA-0.5 had fewer pores in its microstructure than HEA-1 and HEA-2, and the pore shape changed from long strips to nearly circular ones. HEA-2 had significantly more pores owing to the longer ball milling duration. The increased number of pores indicates that the oxygen content of HEA-2 (3.08 wt. %) was higher than that of HEA-1 (1.73 wt. %) and HEA-0.5 (1.55 wt. %). Nevertheless, the number and size of pores in the alloy samples after ball milling were significantly reduced compared to those in the coarsely mixed samples. Regardless of ball milling duration, HEA-0.5, HEA-1, and HEA-2 showed a BCC1 and HCP multiphase structure, with two phases of dark gray HCP phase aggregated by Zr and Hf and light gray BCC1 phase formed by Ti, Ta, and Nb. Additionally, the number of HCP phases increased, and size decreased with longer ball milling time.

The differences between the microstructure of the alloys and the XRD pattern can be attributed to the metal element’s initial particle size and melting point. The particle size of the powder in the rotating mixed samples did not change. In the raw material testing, the maximum particle size of metal powder, especially NbH_2_ and ZrH_2_, was above 30 μm. The melting points of Ta and Nb were the highest among the five elements (above 2800 °C). Consequently, the diffusion rate of Ta and Nb remained low during sintering at 1000 °C, significant diffusion did not occur, and initial aggregation largely remained after sintering. At a 1000 °C sintering temperature, all samples did not show significant element aggregation. For HEA-1, the longer ball milling time and brittle metal hydride provided a finer powder, which enhanced the diffusivity between alloying elements. This enhanced diffusivity led to less aggregation of bulk elements and a more uniform element distribution. As the same time, the long-time ball milling results in serious oxygen pollution, and the strong affinity of metal during the sintering process, so the appearance of Me_x_O_y_ phase cannot be avoided.

**Figure 7:**
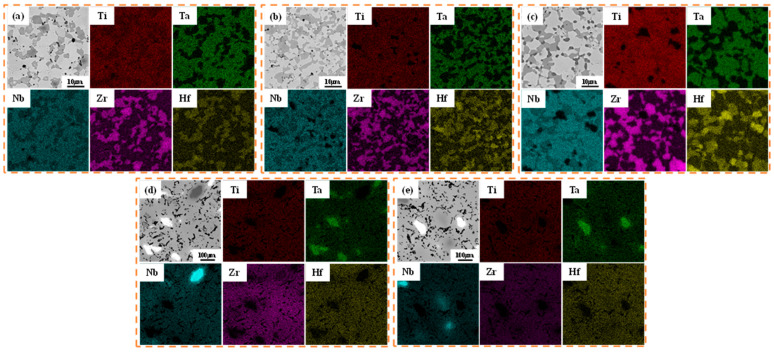
Elemental distributions of the TiNbTaZrHf HEAs at 1400 °C: (**a**) HEA-0.5; (**b**) HEA-1; (**c**) HEA-2; (**d**) HEA-150; (**e**) HEA-300.

**Figure 8:**
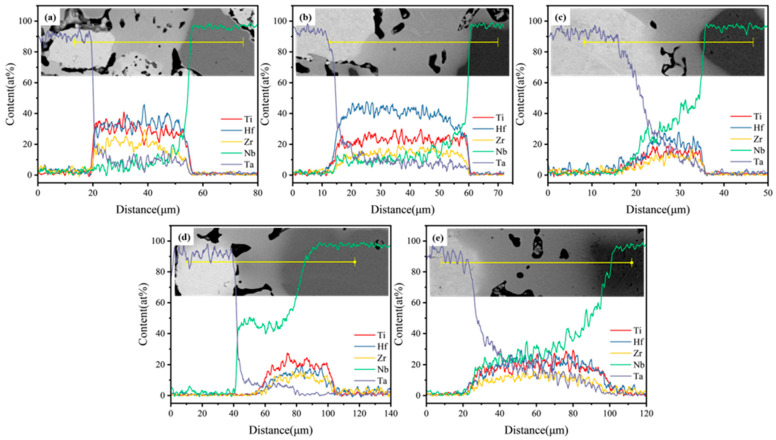
EDS line spectrum performed om HEA-300 at different temperatures showing SEM image with the location of the line and the chemical composition of the HEA-300s: (**a**) 1000 °C; (**b**) 1100 °C; (**c**) 1200 °C; (**d**) 1300 °C; (**e**) 1400 °C.

The factors hindering the diffusion of elements at low temperatures were primarily powder particle size and melting point. However, to date, the effects of sintering temperature on an alloy’s diffusivity remain unknown. Figure 7 illustrates the alloy’s compositional analysis and elemental distribution at 1400 °C. When compared with a lower sintering temperature of 1000 °C, alloys such as HEA-1 possess an apparent two-phase distribution, and the element distribution is more prominent. Meanwhile, HEA-150 and HEA-300 are a single BCC2 phase with a uniform element distribution, indicating that higher sintering temperatures promote element diffusion. Taking HEA-300 as an example, element diffusion analysis was obtained using an EDS line scan. The EDS line spectrum of HEA-300 was performed on dark, light, and BCC2 phases (Figure 8). Figure 8 reveals that different sintering temperatures affected the diffusion of elements in the HEA-300 alloy in that the diffusion of refractory elements Ta and Nb increased as the sintering temperature increased. The EDS line scan results show that when sintering at a lower temperature, the boundary between the Ta and Nb particles and the BCC2 phase was clear. In addition, the element strength detected in the BCC2 phase presented a cliff-like descent. At higher sintering temperatures, Ta and Nb diffused to the surrounding phases, and the strength of the elements decreased slowly. Thus, EDS analysis of HEA-300 confirmed that increasing the sintering temperatures will accelerate the diffusion of Ta and Nb. 

**Figure 9:**
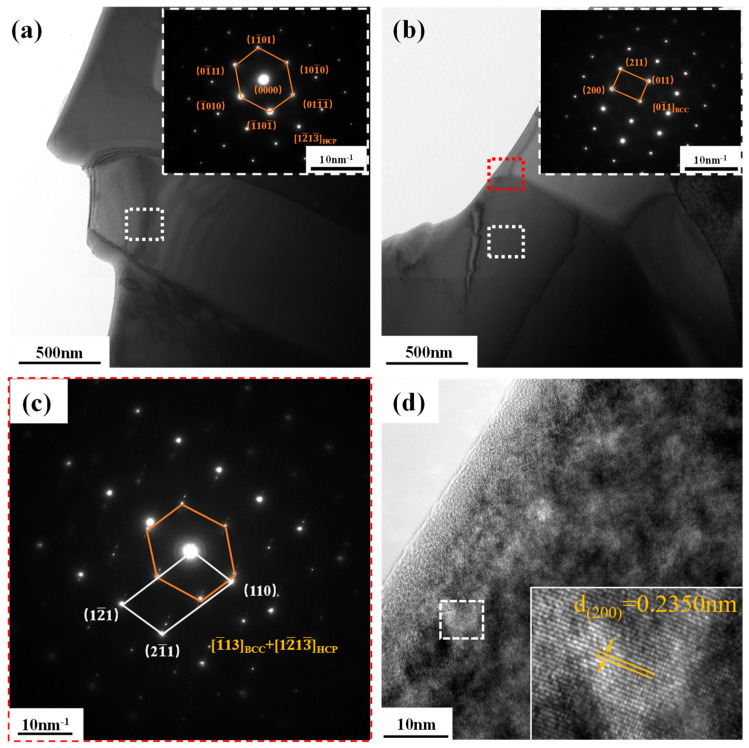
Detailed phase structure obtained by TEM: (**a**) the image of the HCP phase in HEA-1 (inserted image gives the corresponding SAED patterns); (**b**) the image of BCC phase in HEA-1 (inserted image shows the corresponding SAED patterns of the white area); (**c**) the image of BCC and HCP phase in HEA-1 (inserted image gives the corresponding SAED patterns of the red area); (**d**) high-resolution TEM image of BCC phase in HEA-1 (inserted image is the magnified view of the marked region).

**Figure 10:**
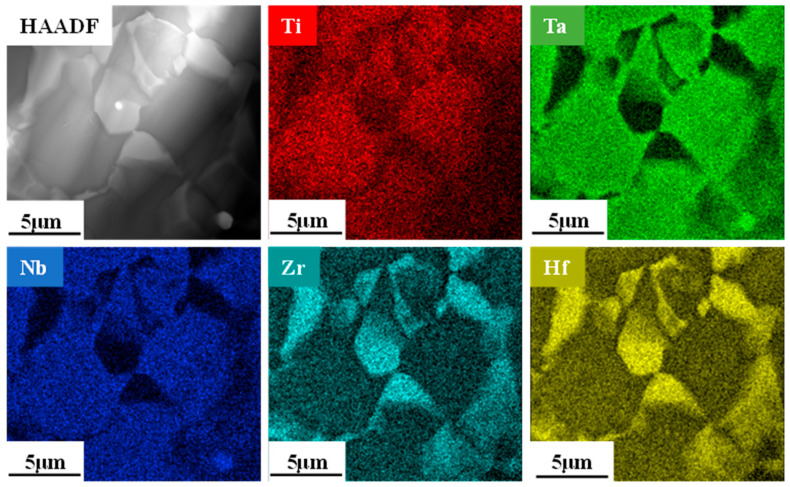
STEM-HAADF image of the as-sintered HEA-1 at 1300 °C.

HEA-150 and HEA-300 demonstrated similar phase structures, and HEA-1 was characterized by complex microstructures. An analysis of the TEM images of HEA-1’s microstructure revealed that its BCC1 phase was a disordered solid solution phase (Figure 9b). The interplanar spacing was measured at d = 0.2350 nm for the crystal plane of (200) (Figure 9d). Figure 9a shows the HCP phase without any precipitate, and the selected area electron diffraction (SAED) patterns verifies the single HCP phase. The red area in Figure 9b demonstrated the boundary between light and dark, and two overlapping electron diffraction spots also appeared in the SAED pattern. The diffraction axes were [$\bar{1}13$]_BCC_ and [1$\bar{2}1\bar{3}$]_HCP_ (Figure 9c). The chemical composition analysis of HEA-1 exhibited a distinct interface between BCC1 and HCP phases without any visible second phase (Figure 9b). The chemical composition also indicated that the HCP phase contained Hf and Zr, and the dark phase in the BCC1 phase was formed by Nb, Ti, and Ta (Figure 10).

### 3.3. Mechanical Properties of the TiTaNbZrHf HEAs

**Figure 11:**
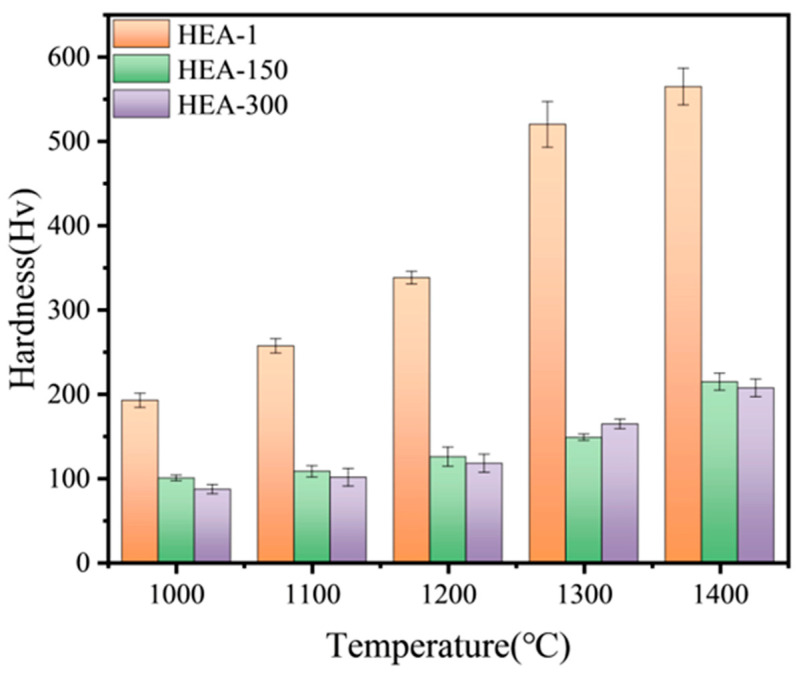
The hardness of the TiTaNbZrHf HEAs.

Figure 11 compares the hardness of samples of different powder particle sizes after sintering at various temperatures. There was no significant difference between the hardness of the coarser HEA-150 and HEA-300 samples at any sintering. The hardness of both HEA-150 and HEA-300 increased with increasing sintering temperature, reaching a maximum hardness of around 200 HV at 1400 °C (Figure 11). In contrast, the sintered sample of HEA-1 displayed a significant increase in hardness when compared to the coarser samples over all sintering temperatures, peaking at approximately 550 HV at 1400 °C. The substantial observed increase in the hardness of the HEA-1 sample compared to that of both the HEA-150 and HEA-300 samples can be attributed to a mosaic of factors, including a smaller powder particle size, a higher element diffusion rate, the Me_x_O_y_ phase, and the formation of BCC1+HCP two-phase structure. At higher sintering temperatures and long-time ball milling, the increased oxygen content of HEA-1 and the subsequent oxide formation particle strengthening played a significant role in the substantial increase in HEA-1’s hardness. Table 1 illustrates the compressive actual stress–strain curve for the alloys at room temperature. The yield strength, compressive strength, and plastic strain of the HEA-1 were 1400 MPa, 1620 MPa, and 20%, respectively. HEA-1 displayed higher yield strength when compared to RHEAs such as as-cast TiZrNbHfTa, TaNbHfZr, and TiZrNbTa in Table 1. In particular, when comparing HEA-1 to the as-cast TiZrNbHfTa, the yield strength was greatly improved and the plastic strain was reduced. The superhigh strength, hardness, and reduced plastic strain of our samples can be associated with oxide formation particle strengthening caused by metal oxide. A similar increase in mechanical properties was reported for TiZrHfNbO alloy [32,33].

## 4. Conclusions

In this study, TiTaNbZrHf HEAs were produced by cold isostatic pressing and pressureless sintering. The alloy powders had different particle sizes, which impacted the microstructure and mechanical properties of the alloys due to the diffusivity of various elements. The alloy obtained under the optimum conditions in the present work exhibited a maximum hardness of 560 HV and a maximum strength of 1620 MPa. The compressive yield strength of the TiTaNbZrHf HEAs fabricated using MA and mixed-atmosphere sintering was significantly higher than that of previously reported HEAs processed by arc melting and casting.

## Figures and Tables

**Table 1:** Room-temperature compressive properties of RHEAs in the references and the present work.

Materials	Preparation Methods	Phase Structure	Yield Strength (MPa)	Plastic Strain (%)
TiZrNbHfTa [28]	As-cast	BCC	929	>50
TaNbHfZr [17]	As-cast	BCC	1315	21.6
TiZrNbTa [34]	As-cast	BCC	1100 ± 90	48 ± 6
TiTaNbZrHf	PM	BCC + HCP	1400	20
Hf_20_Nb_10_Zr_35_Ti_35_ [35]	As-cast	BCC	524	17.5
TiTaNbZrHf [36]	PM	BCC	940	>50
TiTaNbZrHf [37]	As-cast	BCC	1000	19
